# Supplementary material for: Whole tissue spatial cellular analysis reveals increased macrophage infiltration in pancreata of autoantibody positive donors and patients with type 1 diabetes
Source: bioRxiv. 2025 Sep 17:2025.09.13.676053. Preprint. [Version 1] doi: 10.1101/2025.09.13.676053 (PMC12458208; doi:10.1101/2025.09.13.676053)
Supplement: Supplement 1 [file media-1.pdf]

Supp. Table 1: Clinical Characteristics of Pancreatic Organ Donors

Disease	nPOD #	Age (years)	BMI (kg/m²)	Sex	Time in ICU (days)	C-peptide (mmol/L)	T1D duration (years)	Autoantibodies
Non-diabetic	6029	24	22.6	F	–	–	–	–
Non-diabetic	6073	19.2	36	M	4.47	0.69	–	–
Non-diabetic	6098	17.8	22.8	M	1.74	1.41	–	–
Non-diabetic	6251	33	29.5	F	3.73	1.92	–	–
Non-diabetic	6271	17	24.4	M	0.48	11.47	–	–
Aab+	6123	23.2	17.6	F	3.55	2.01	–	GADA
Aab+	6147	23.8	32.9	F	2.65	3.19	–	GADA
Aab+	6167	37	26.3	M	2.95	5.43	–	IA2A, ZnT8A
Aab+	6197	22	28.2	M	2.96	17.48	–	GADA, IA2A
Aab+	6301	26	32.08	M	3.16	3.92	–	GADA
T1D	6039	28.7	23.4	F	–	<0.05	12	GADA, IA2A, mIAA, ZnT8A
T1D	6084	14.2	26.3	M	2.48	<0.05	4	mIAA
T1D	6198	22	23.1	F	21.71	<0.05	3	GADA, IA2A, mIAA, ZnT8A
T1D	6380	11.6	14.6	F	4.02	0.22	0	–
T1D	6399	17.42	32	M	–	1.41	0	GADA, IA2A, ZnT8A

Aab+, autoantibody-positive; F, female; GADA, GAD autoantibodies; IA2A, insulinoma-associated protein 2 autoantibodies; M, male; mIAA, micro-insulin autoantibodies; nPOD #, nPOD identification number; T1D, type 1 diabetes; ZnT8A, zinc transporter 8 autoantibodies.

Supp. Fig. 1: Visual representation of regional and spatial cell distribution analysis methodology

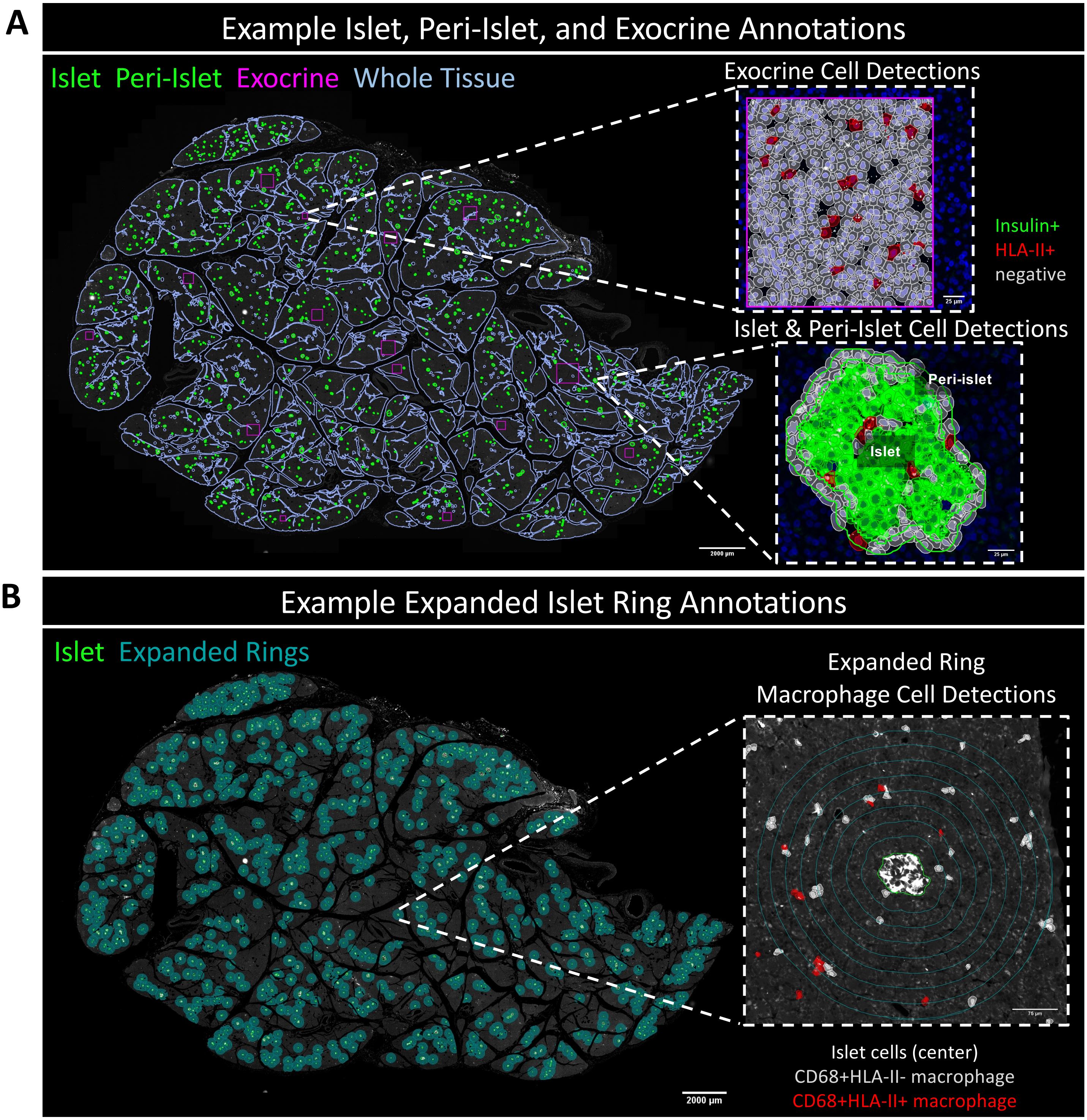

The spatial distribution of macrophages within each pancreatic tissue section was analyzed with two different semi-automated methods using machine learning algorithms and custom groovy scripts in QuPath. **(A)** Whole tissue annotation (purple). Regional analysis with automatically detected islet (green) and peri-islet (green; 10 µm radius) regions and selected exocrine regions (pink). Both amplified images show the cell classification in regions of interest. **(B)** Islet proximity analysis with automatically detected islet regions (green) and expanded islet regions consisting of 8 concentric rings (teal; 25 µm radius). For both methods, the number, percentage and density of cells were counted within each region and categorized by their classification (positive or negative) for each marker (insulin, CD68, and HLA class II).

Supp. Fig. 2: Macrophage infiltration is greatest in the pancreas of donors with type 1 diabetes regardless of region

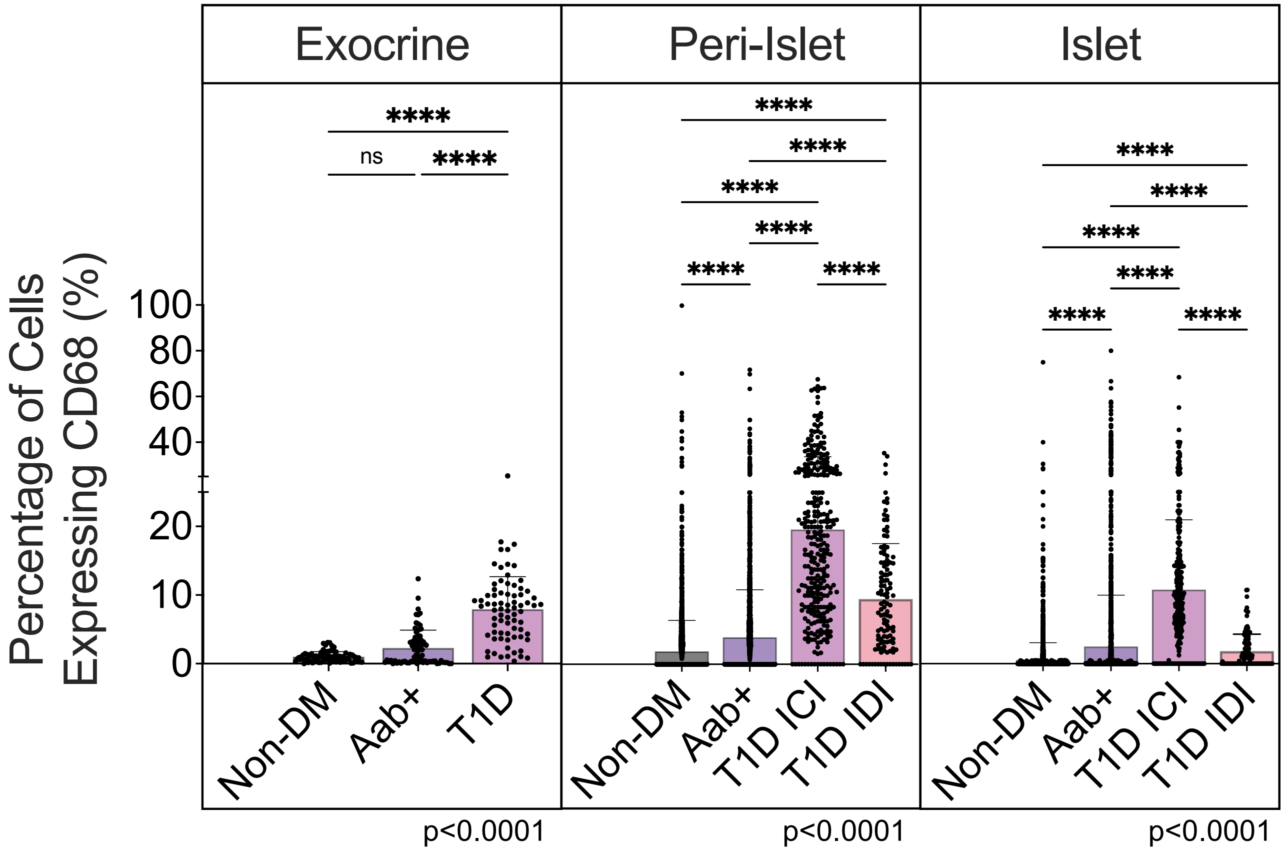

The percentage of total cells which express CD68 is higher in the insulin-containing islets (ICI) and associated peri-islet (10 µm radius) and exocrine regions of the T1D cases than any other group. The differences observed between groups were statistically significant for the exocrine, peri-islet and islet regions ($p<0.0001$). Each datapoint represents one region (i.e., one islet), and the mean + SD is shown. Statistical significance was calculated using Kruskal-Wallis test, and multiple comparisons were assessed using Dunn's test (**** $p<0.0001$, ns not-significant).

Supp. Fig. 3: Macrophage distribution near islets varies within and between groups

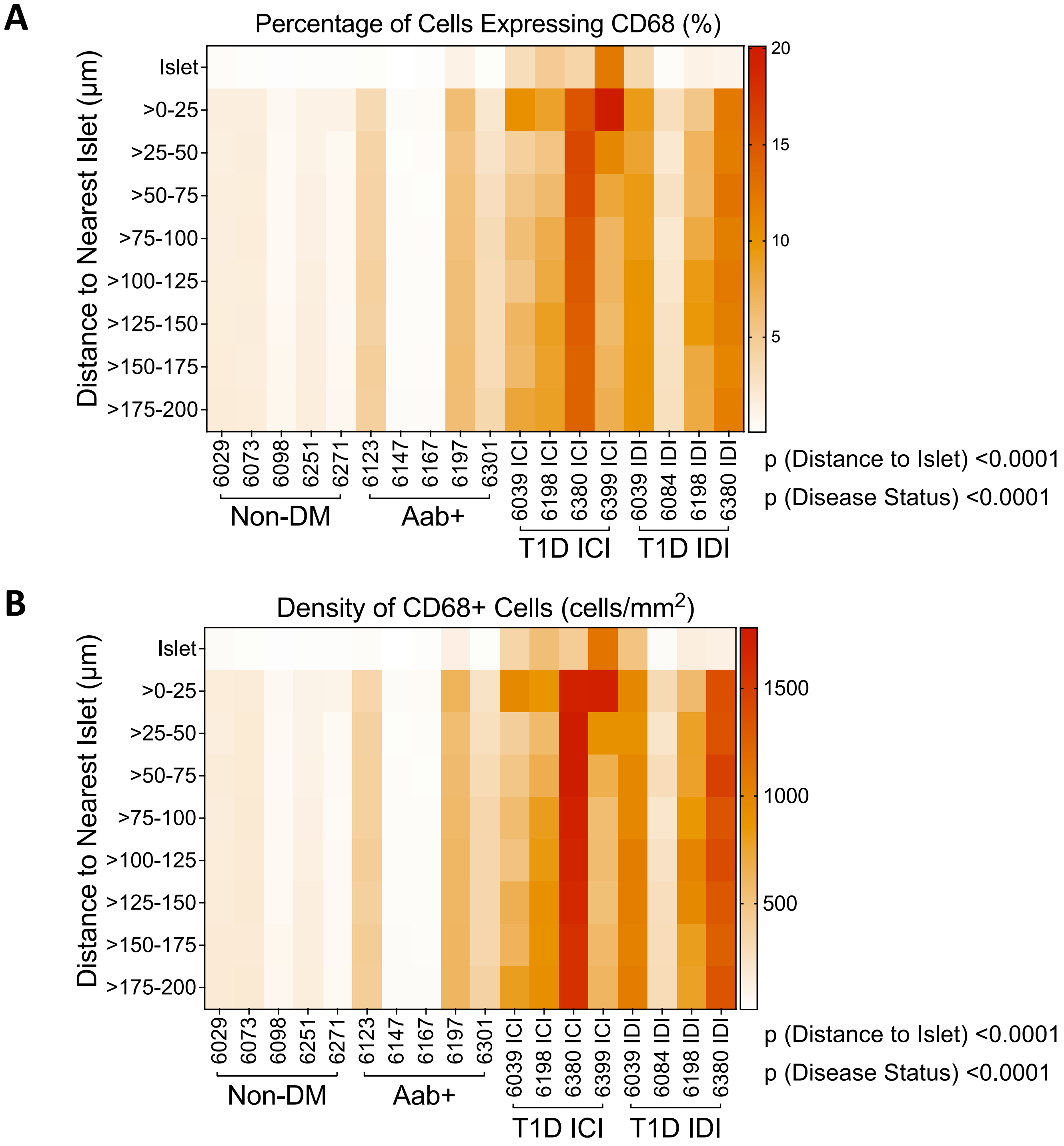

Case-wise representation of the expanded islet region analysis consisting of 8 concentric rings (25 µm radius). Both the **(A)** percentage and **(B)** density of cells expressing CD68 within each ring varied between cases and groups. Statistical significance was calculated using Two-way ANOVA test, and multiple comparisons were assessed using either Tukey's test.

Supp. Fig. 4: Distance-wise variation of macrophage distribution for expanded ring spatial analysis

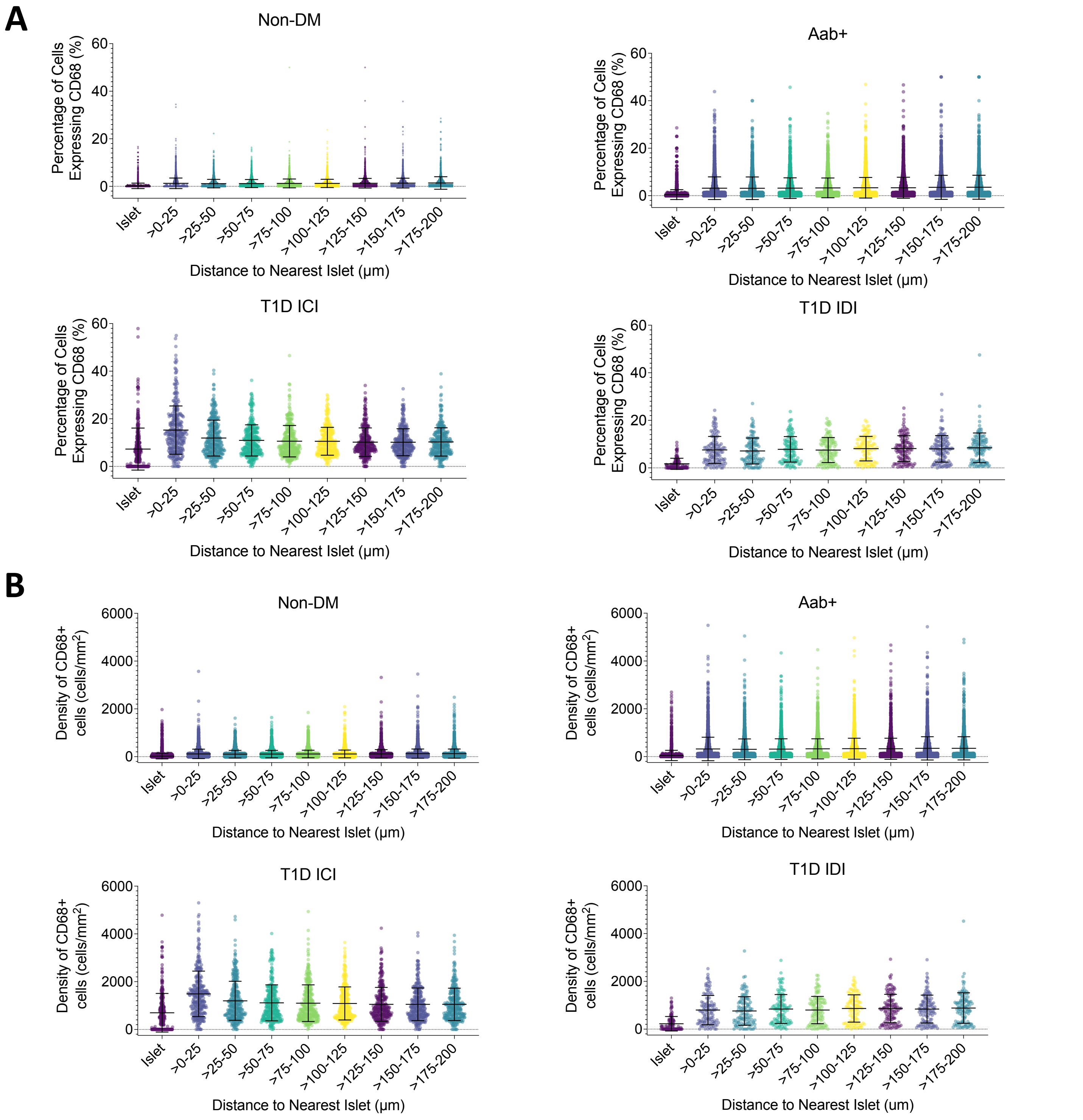

Raw data of the expanded islet region analysis consisting of 8 concentric rings (25 µm radius) for the **(A)** percentage and **(B)** density of cells expressing CD68 within each ring. Each datapoint represents one region and the mean ± SD is shown.
